# Higher Muscle Mass and Higher Serum Prealbumin Levels Are Associated with Better Survival in Hemodialysis Patients during a Five-Year Observation Period

**DOI:** 10.3390/nu15051237

**Published:** 2023-02-28

**Authors:** Anna Jeznach-Steinhagen, Iwona Boniecka, Aleksandra Rymarz, Monika Staszków, Jerzy Romaszko, Aneta Czerwonogrodzka-Senczyna

**Affiliations:** 1 Department of Clinical Dietetics, Faculty of Health Sciences, Medical University of Warsaw, E Ciołka 27, 01-445 Warsaw, Poland; 2 Department of Internal Medicine, Nephrology and Dialysis, Military Institute of Medicine, Szaserów 128, 04-141 Warsaw, Poland; 3 Department of Nephrology, Dialysis and Internal Medicine, Medical University of Warsaw, Banacha 1 A, 02-097 Warsaw, Poland; 4 Department of Family Medicine and Infectious Diseases, University of Warmia and Mazury in Olsztyn, Warszawska 30, 10-082 Olsztyn, Poland

**Keywords:** muscle mass, hemodialysis, prealbumin, malnutrition

## Abstract

Background: Dialysis is the most commonly used renal replacement therapy in patients with end-stage renal disease. The mortality rate of hemodialysis patients is 15–20%, with cardiovascular complications being the most common. There is an association between the severity of atherosclerosis and both the development of protein-calorie malnutrition and inflammatory mediators. The aim of this study was to assess the relationship between biochemical markers of nutritional status, body composition and survival in hemodialysis patients. Methods: Fifty-three hemodialysis patients were included in the study. Serum albumin, prealbumin, and IL-6 levels were measured, as well as body weight, body mass index, fat content and muscle mass. The five-year survival of patients was calculated using Kaplan–Meier estimators. The long-rank test was used for univariate comparison of survival curves, and the Cox proportional hazards model was used for multivariate analysis of survival predictors. Results: There were 47 deaths, 34 of which were due to cardiovascular disease. The hazard ratio (HR) for age in the middle-aged group (55–65 years) was 1.28 (confidence interval [CI] 0.58, 2.79) and 5.43 (CI 2.1, 14.07; statistically significant) for the oldest age group (over 65 years). A prealbumin level above 30 mg/dl was associated with an HR of 0.45 (CI 0.24, 0.84). Serum prealbumin (odds ratio [OR] = 5.23; CI 1.41, 19.43; *p* = 0.013) and muscle mass (OR = 7.5; CI 1.31, 43.03; *p* = 0.024) were significant predictors of all-cause mortality. Conclusions: Prealbumin level and muscle mass were associated with increased mortality risk. Identification of these factors may improve the survival of hemodialysis patients.

## 1. Introduction

Worldwide, the total number of individuals with acute kidney injury, CKD and Renal Replacement Therapy (RRT) exceeds 850 million, a figure that is double the estimated number of people with diabetes worldwide [1]. Data from the 2017 Annual Report of the European Renal Association—European Dialysis and Transplant Association (ERA-EDTA) [2] report that 83,311 individuals from all countries of Europe started RRT in 2016. At the end of the same year, the total number of individuals requiring RRT was 564,638, with more than 80% being on hemodialysis (HD).

Dialysis is the most common renal replacement therapy for patients with end-stage renal disease. This paper considers the survival of dialysis patients and aims to assess both anthropometric and biochemical nutritional parameters. Currently, there are over 21,000 hemodialysis patients in Poland. The mortality rate for hemodialysis patients is 15–20%, which is four to seven times higher than for the general population. The most common complications in this group of patients are cardiovascular complications, which cause more than 50% of deaths [3]. A relationship exists between the severity of atherosclerosis and both the development of cardiovascular complications and mediators of inflammation, such as the concentration of proinflammatory cytokines (MIA syndrome: malnutrition, inflammation, atherosclerosis). The presence of MIA in dialysis patients is the cause of the reverse epidemiology of cardiovascular disease (CVD). Factors that reduce the risk of CVD in the general population increase the likelihood of the occurrence and development of these diseases, and of death, in patients who are dialyzed.

Hemodialysis (HD) majorly represents the global treatment option for patients with chronic kidney disease at stage 5, and, despite advances in dialysis technology, these patients face a high risk of morbidity and mortality from malnutrition. Sahathevan et al. [4] aimed to provide a novel view that malnutrition susceptibility in the global HD community is either or both of iatrogenic and non-iatrogenic origins. This categorization of malnutrition origin clearly describes the role of each factor in contributing to malnutrition. Low dialysis adequacy resulting in uremia and metabolic acidosis and dialysis membranes and techniques, which incur greater amino-acid losses, are identified as modifiable iatrogenic factors of malnutrition. Dietary inadequacy as per suboptimal energy and protein intakes due to poor appetite status, low diet quality, high diet monotony index, and/or psychosocial and financial barriers are modifiable non-iatrogenic factors implicated in malnutrition in these patients. These factors should be included in a comprehensive nutritional assessment for malnutrition risk. Leveraging the point of origin of malnutrition in dialysis patients is crucial for healthcare practitioners to enable personalized patient care, as well as determine country-specific malnutrition treatment strategies.

A meta-analysis of sub-Saharan African HD patients shows insufficient infrastructure and catastrophic out-of-pocket costs. Most patients remain undiagnosed, untreated, and die. In the pooled analysis, 390 (96%) of 406 adults and 133 (95%) of 140 children who could not access dialysis died or were presumed to have died. Among those dialyzed, 2747 (88%) of 3122 adults in incident ESKD cohorts, 496 (16%) of 3197 adults in prevalent ESKD cohorts, and 107 (36%) of 294 children with ESKD died or were presumed to have died. Further to this, 2508 (84%) of 2990 adults in incident ESKD cohorts discontinued dialysis compared with 64 (5%) of 1364 adults in prevalent ESKD cohorts, and 41 (1%) of 4483 adults in incident ESKD cohorts, 2280 (19%) of 12 125 adults in prevalent ESKD cohorts, and 71 (19%) of 381 children with ESKD received transplants. Sixteen studies reported on the management of anemia, 17 on dialysis frequency, eight on dialysis accuracy, and 22 on vascular access for dialysis. Most patients with ESKD starting dialysis in sub-Saharan Africa discontinue treatment and die. Further work is needed to develop equitable and sustainable strategies to manage individuals with ESKD in sub-Saharan Africa [5].

Malnutrition is a common issue among hospitalized patients [6]. Malnutrition in the chronic dialyzed patient has been a serious clinical problem for a long time and still occurs in about one-third of these patients, ranging from 20–76%, according to various sources [6,7,8]. Two types of malnutrition are observed in patients with kidney disease, both of which may lead to accelerated development of the MIA syndrome. The first is associated with an insufficient supply of protein and with energy and protein absorption disorders. The second is associated with chronic inflammation [9]. Inflammation, manifested by an increase in C-reactive protein (CRP) and proinflammatory cytokine levels in the serum, is an important cause of malnutrition. Interleukin (IL)-6 plays a significant role in the development of malnutrition through the catabolism of muscle proteins and the anorectic effect of cytokines (increased leptin production and lipolysis). An increased level of cytokines is characteristic of hypoalbuminemic patients and is associated with a shorter survival [10,11].

Sarcopenia in end-stage kidney disease patients requiring dialysis is a frequent complication but remains an under-recognized problem.

The Wathanavasin et al. (2022) meta-analysis was conducted to determine the prevalence of sarcopenia and explore its impacts on clinical outcomes, especially cardiovascular events and mortality in dialysis patients. The eligible studies were searched from PubMed, Scopus, and Cochrane Central Register of Controlled trials up to 31 March 2022. The result showed that forty-one studies with 7576 patients were included. The pooled prevalence of sarcopenia in dialysis patients was 25.6% (95% CI 22.1 to 29.4%). Sarcopenia was significantly associated with higher mortality risk (adjusted OR 1.83 (95% CI 1.40 to 2.39)) and cardiovascular events (adjusted OR 3.80 (95% CI 1.79 to 8.09)). Additionally, both low muscle mass and low muscle strength were independently related to increased mortality risk in dialysis patients (OR 1.71; 95% CI (1.20 to 2.44), OR 2.15 (95% CI 1.51 to 3.07)), respectively. This meta-analysis revealed that sarcopenia was highly prevalent among dialysis patients and has been shown to be an important predictor of cardiovascular events and mortality [12].

Progressive nutritional impairment has been recently reported during conventional hemodialysis (HD) treatment. The Chasot (2006) study showed that the nutritional parameters during a five-year follow-up in HD patients were stable during the five-year period. Thirty-three patients (15F/18M; 65 years old at the study start) filled out a three-day food questionnaire once a year between 1995 and 1999 (study group). Twenty patients who did not fill out the food records during this period served as a control group (control group). The food record was run by the renal dietician using dedicated software, providing daily energy and protein intakes. Serum albumin, normalized protein equivalent of nitrogen appearance (nPNA), and post-dialysis body weight (BW) at the time of food record were collected in the study group and from the patient chart in the control group. The energy intake in the study group and the protein intake in both groups were close to the recommended intakes in ESRD patients. Protein intake assessed from food questionnaires or from urea kinetics was not statistically different. Using ANOVA for repeated measures, no difference along the five years was found for daily energy intake, daily protein intake, nPNA, and BW in the study group. The BW and nPNA remained stable in the control group. Hence, this study does not confirm the progressive nutritional impairment reported in the HEMO study, whereas the patients’ age and vintage are largely higher in the present study [13].

The inflammatory process in hemodialysis patients plays a key role in the pathogenesis of atherosclerosis and CVD. Causes of inflammation in patients with kidney disease include increased oxidative stress, hypertension, subclinical infections, accumulation of metabolism products, endotoxin effects, hypercatabolism, and genetic factors. Around 35–65% of hemodialyzed patients experience chronic inflammation [14]. Already in the early stages of chronic renal failure, patients with no heart disease have an increased level of proinflammatory cytokines (IL-1, TNF-α, and IL-6), acute phase proteins (CRP and fibrinogen), adhesion molecules (selectins), and some blood coagulation factors, as well as reduced expression of anti-inflammatory cytokines. The level of IL-6 increases as the globular filtration rate decreases and is a strong predictor of poor prognosis. Increased serum levels of IL-6 and CRP predispose people without kidney disease to the development of CVD and increased mortality. A high concentration of IL-6 in patients initiating renal replacement therapy is associated with a worse distant prognosis [15].

Identifying risk factors for mortality may help in early intervention approaches to improve the survival of patients on chronic hemodialysis who have a substantially reduced life expectancy.

## 2. Materials and Methods

We conducted a single-center study in the Dialysis and Internal Diseases Clinic of the Medical University of Warsaw. The study followed 53 patients (20 women and 33 men). The mean (SD) age of patients was 58.6 (5.6) years. They had chronic renal failure and received hemodialysis regularly since December 2005.

### 2.1. Blood and Anthropometric Parameters

The levels of plasma albumin, prealbumin, and IL-6, as well as the weight, body mass index (BMI, calculated as weight/height^2^), fat content, and muscle mass of all patients, were measured at the beginning of the study. To be included in the study, patients had to have undergone hemodialysis regularly and been clinically stable, without any nutrition support, for at least three months before being enrolled in the study.

The anthropometric parameters of nutritional status, including body weight, BMI, skinfold thickness over the triceps muscles, mid-upper arm circumference, and waist and hips circumference, were evaluated after dialysis.

Body weight and fat content (fat %) were measured with high-quality electronic calibrated scales Tanita TBF 300P (to the nearest 0.1 kg), and height was measured with a wall-mounted stadiometer SECA 216 (to the nearest 0.5 cm). Body mass index (BMI) was calculated as weight in kilograms divided by the square of height in meters.

Waist circumference was measured at the midpoint between the lower margin of the least palpable rib and the top of the iliac crest using a stretch-resistant tape. Hip circumference was measured around the widest portion of the buttocks, with the tape parallel to the floor. The waist-hip ratio (WHR) was calculated by dividing waist circumference by hip circumference [16].

Mid-upper arm circumference was measured (to the nearest 0.1 cm) using a flexible nonstretch tape laid at the midpoint between the acromion and olecranon processes on the shoulder blade and the ulna, respectively, of the arm [17].

The triceps skin fold thickness was measured using a Harpenden skinfold caliper.

The results were compared to the standards in Table 1.

Blood was collected immediately before dialysis. The biochemical determinations were performed on a Cobas Integra 800 analyzer (Roche). The IL-6 cytokine assay was performed using the Fluorokine MAP kit cytokine multiplex kit (R&D Systems) and the Luminex 100 ™ instrument.

Patients were under the constant care of the center. The attending physician had access to the patients’ full documentation, on the basis of which the date and cause of death were established.

The study was approved by the Bioethics Committee of the Medical University of Warsaw. All patients signed an informed consent form before the beginning of the study.

### 2.2. Statistical Analysis

The five-year patient survival was computed using Kaplan–Meier estimates. Univariate comparison of survival curves was performed using the log-rank test. A multivariate analysis of survival predictors was done using Cox proportional hazards model. The multivariate model was built using stepwise variable selection based on Akaike Information Criterion. Results are expressed as hazard ratios (HR) with 95% confidence intervals (CI). A logistic regression analysis for the endpoint, defined as survival of more than five years, was performed. Results are expressed as the odds ratio (OR) with its 95% CI. T-tests were used to compare patients living shorter and longer than five years. The dependence between selected descriptive variables was described using a linear correlation coefficient matrix. The statistical analysis was carried out using R 3.5.1 statistical software (R Core Team 2018). Statistical significance was considered at *p* values < 0.05.

## 3. Results

The characteristics of the study group, including the mean age, length of follow-up, and hemodialysis vintage, are presented in Table 2. Patients who were switched over to other forms of renal replacement therapy, like renal transplantation, after inclusion in the study were excluded (*n* = 3). A total of 50 patients fulfilled the inclusion criteria and were included in the analysis.

The mean follow-up time was 13 years, during which there were 47 deaths: 34 due to CVD and 13 due to sepsis. The most common causes of death were ischemic heart disease and pulmonary embolism, both accounting for nearly 72% of the overall mortality.

**Figure 1:**
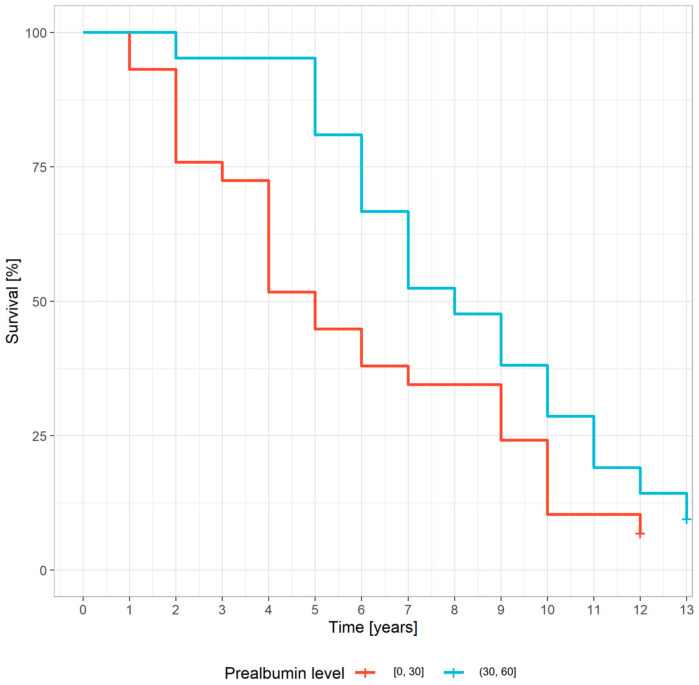
The relationship between serum prealbumin concentration and survival (Kaplan–Meier estimates) (the blue and red lines represent prealbumin concentration ranges [mg/dL]).

**Figure 2:**
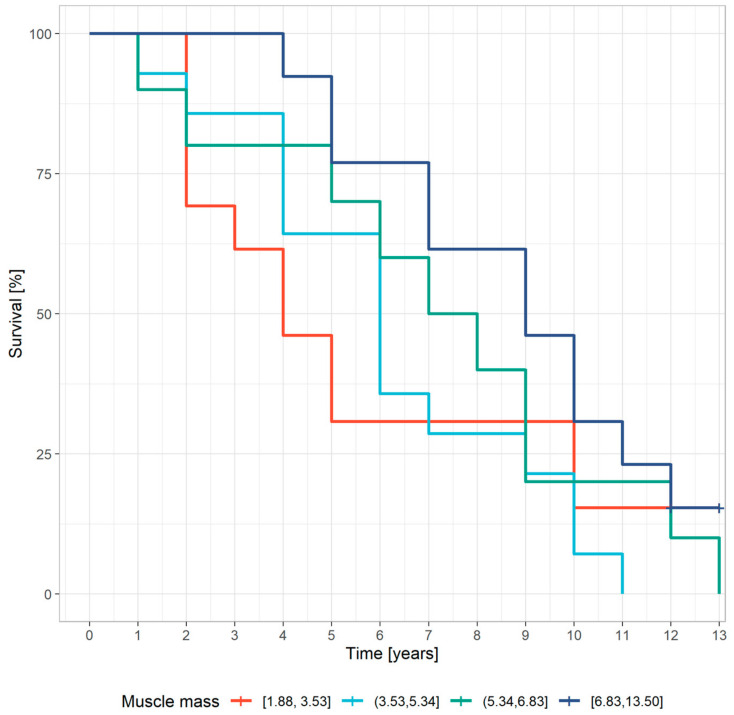
The relationship between muscle mass and survival (Kaplan–Meier estimates) (the colors of the lines indicate ranges of muscle mass content [cm^3^]).

A statistically significant relationship was observed between the five-year survival of patients and the prealbumin concentration and muscle mass (Table 3, Figure 1 and Figure 2).

In univariate analysis, the HR for age in the middle-aged group (55–65 years) was 1.28 (0.58, 2.79). For the oldest age group (over 65 years), the HR for age was 5.43 (2.1, 14.07; statistically significant). A prealbumin level over 30 mg/dL was associated with an HR of 0.45 (0.24, 0.84) (Table 4).

A crude analysis showed that serum prealbumin (SPA; OR = 5.23 [1.41, 19.43], *p* = 0.013) and muscle mass (OR = 7.5 [1.31, 43.03], *p* = 0.024) were significant predictors of all-cause mortality (Table 5).

## 4. Discussion

In this study, the amount of muscle mass rather than BMI was associated with survival in hemodialysis patients. Previous studies considered BMI as one of the markers of nutrition status capable of influencing survival rate. Unlike that of the general population, the survival rate of hemodialysis patients decreases as the BMI increases. A similar trend was described in patients with chronic obstructive pulmonary disease and heart failure [18,19]. This phenomenon is called reverse epidemiology or survival paradox. One of the explanations is that the BMI does not reflect body composition. In other words, the same BMI can be associated with different amounts of muscle and fat mass. The loss of muscle mass resulting from unfavorable metabolic disorders seems to be a sensitive predictor of mortality in the short term. Sarcopenia, the loss of muscle mass, can be masked by an increase in fat mass, which would not be reflected in the BMI. Sarcopenic obesity is reached when muscle mass decreases but fat mass increases, elevating the BMI to the level of obesity. This phenotype is associated with greater cardiovascular and all-cause mortality [20,21].

Ebrahimi et al.’s study [22] evaluated the effect of different factors on the survival time of these patients. In this study, parametric survival models were used to find the factors affecting survival and discover their effect on survival time. Of 428 HD patients eligible for the analysis, 221 (52%) experienced death. With the mean ± SD age of 60 ± 16 years and BMI of 23 ± 4.6 kg/m, they comprised 250 men (58%). The median survival time (95% CI) was 624 days (550 to 716). The overall 1, 2, 3, and 4-year survival rates for the patients undergoing HD were 74, 42, 25, and 17%, respectively. By using AIC, AFT log-normal model was recognized as the best functional form of survival time. Cox-adjusted PH results showed that the amount of ultrafiltration volume (UF) (HR = 1.146, *p* = 0.049), WBC count (HR = 1.039, *p* = 0.001), RBC count (HR = 0.817, *p* = 0.044), MCHC (HR = 0.887, *p* = 0.001), and serum albumin (HR = 0.616, *p* < 0.001) had significant effects on mortality. AFT log-normal model indicated that WBC (ETR = 0.982, *p* = 0.018), RBC (ETR = 1.131, *p* = 0.023), MCHC (ETR = 1.067, *p* = 0.001), and serum albumin (ETR = 1.232, 0.002) had a significant influence on the survival time.

The results of our study correspond with these observations. A higher amount of muscle mass was associated with a better survival rate, whereas the BMI did not influence survival (*p* = 0.1). The likelihood of surviving more than five years increased in each higher muscle mass quartile and was the highest for patients with the greatest muscle mass. The OR for the fourth quartile of muscle mass was 7.5 in comparison with the first quartile. Fukasawa et al. noted that lower tight muscle mass measured by computer tomography was associated with an increased all-cause and cardiovascular mortality in hemodialysis patients [23]. A lower lean tissue index, an equivalent of muscle mass measured by bioimpedance spectroscopy, also correlated with a worse survival rate in dialysis patients in recent studies [21,22,23,24,25,26,27]. A reduced muscle mass was also a predictor of mortality in individuals without kidney failure. Srikanthan et al. observed the lowest mortality rate in the highest muscle mass quartile in patients with cardiovascular disease [28].

In chronic kidney disease (CKD) patients, muscle mass loss can be caused by many factors, including inflammation. CKD is a low-grade inflammatory state, and elevated levels of cytokines such as IL-6, Il-1β, and TNF-α are observed in CKD patients [29,30]. Inflammation activates catabolic processes, which result in muscle degradation through the activation of the ubiquitin-proteasome system and insulin resistance. Consequently, a higher mortality rate is observed in patients with elevated levels of these cytokines and concomitant CKD [31,32]. Nonetheless, in our study, the serum level of IL-6 was not a statistically significant predictor of mortality (*p* = 0.9).

Among the biochemical markers of nutrition that can define protein-energy wasting are serum albumin, prealbumin, and cholesterol levels [17,18]. The cut-off point for protein-energy wasting for serum albumin is 3.8 g/dL and 30 mg/dL for serum prealbumin (SPA). Our results revealed a significant association between the SPA level and mortality. Patients with a SPA level higher than 30 mg/dL had a higher survival rate compared with patients with a lower SPA concentration (OR = 5.23, *p* = 0.013). This result is related to the previously described association between muscle mass and mortality. Lean tissue mass mostly made up of muscle, is an important somatic protein store. If a decrease in muscle mass is associated with a higher mortality rate, a reduction in the SPA level can also affect survival. The same trend was described by Rambod et al. [32]. They observed an increased mortality rate in hemodialysis patients that had a SPA level lower than 20 mg/dL and in whom SPA concentrations decreased over 6 months [29]. Furthermore, the SPA level correlated with the amount of muscle mass. Others have also observed this relationship. Kamijo et al. observed a positive correlation between the SPA level and the amount of muscle mass in peritoneal dialysis patients [33].

Ferreira et al. [34] suggest that the optimal management of end-stage renal disease (ESRD) in hemodialysis (HD) patients should be studied more because it is a serious risk factor for mortality, being considered an unquestionable global priority. They performed a retrospective cohort study from the Nephrology Service in Brazil evaluating the survival of patients with ESRD in HD for 20 years to explore the association between survival time and demographic factors, quality of treatment and laboratory values. Data from 422 patients were included. The mean survival time was 6.79 ± 0.37. The overall survival rate in the first year was 82.3%. The survival time correlated significantly with clinical prognostic factors. Prognostic analyses with the Cox proportional hazards regression model and Kaplan–Meier survival curves further identified that leukocyte count (HR = 2.665, 95% CI: 1.39–5.12), serum iron (HR = 8.396, 95% CI: 2.02–34.96), serum calcium (HR = 4.102, 95% CI: 1.35–12.46) and serum protein (HR = 4.630, 95% CI: 2.07–10.34) as an independent risk factor for the prognosis of survival time, while patients with chronic obstructive pyelonephritis (HR = 0.085, 95% CI: 0.01–0.74), high ferritin values (HR = 0.392, 95% CI: 0.19–0.80), serum phosphorus (HR = 0.290, 95% CI: 0.19–0.61) and serum albumin (HR = 0.230, 95% CI: 0.10–0.54) were less risk to die. Survival remains low in the early years of ESRD treatment. The present study identified elevated values of ferritin, serum calcium, phosphorus, albumin, leukocyte, serum protein and serum iron values as useful prognostic factors for survival time. In our study, the most important parameters were: prealbumin and anthropometric data.

The SPA level is also a predictor of mortality in patients with diseases other than CKD. A low prealbumin level was also associated with a higher short-term mortality rate in patients with acute heart failure [35].

A limitation of this study is the small sample size, and therefore, the results should be interpreted with caution.

Anthropometric measurements are simple and inexpensive methods of assessing nutritional status, but their limitation is that they can be influenced by external factors (hydration). If they are not performed on the same equipment and at the same time of day, and by the same person, they are less reproducible. Biochemical markers of malnutrition in our study were analyzed separately. Due to the fact that a correlation between SPA and muscle mass content was shown, it was analyzed only in relation to this parameter. In addition, SPA was found to be a significant predictor of all-cause mortality. We also wanted to provide evidence that even if a determination of prealbumin levels is impossible, the survival prognosis can be estimated based only on anthropometric measurements. Identification of risk factors for mortality can assist in early intervention to improve the survival of patients on chronic hemodialysis who have a significantly shorter life expectancy.

## 5. Conclusions

In our study, a decreased serum albumin level did not influence survival. Muscle mass, rather than BMI, was a statistically significant predictor of survival in hemodialysis patients. The highest muscle mass quartiles were associated with better survival. Among the biochemical markers of nutrition, the SPA level can be a sensitive predictor of mortality. A SPA higher than 30 mg/dL seems to be beneficial.

## Figures and Tables

**Table 1:** Anthropometric measurement standards.

Parameter	Norm
BMI [10]	<18.5 kg/m^2^ underweight 18.5–20 kg/m^2^ normal 25.1–30 kg/m^2^ overweight 30.1–40 kg/m^2^ obesity >40 kg/m^2^ extreme obesity
the triceps skin fold thickness [11]	women 16.5 mm men 12.5 mm
mid-upper arm circumference [11]	women 28.5 cm men 29.3 cm
muscle mass [11]	women 23.2 cm^2^ men 25.3 cm^2^
fat % [11]	women 20–35% men 10–20%
WHR [10]	women < 0.8 men < 0.95
albumin [11,12]	35–55 mg/dL
prealbumin [11,12]	20–40 mg/dL
IL-6 [vs. control group]	0.96 pg/mL

BMI: body mass index; WHR: waist-to-hip ratio; IL-6: interleukin-6.

**Table 2:** Characteristics of the study group (*n* = 53).

Parameter	Mean (±SD)/%
age [years]	58.50 (±5.60)
sex [%] female male	37.70 62.30
renal failure [%] chronic glomerulonephritis diabetic nephropathy chronic pyelonephritis reflux nephropathy nephrosclerosis other	37.70 17.00 15.10 7.50 7.50 15.10
body mass [kg]	69.30 (±16.05)
BMI [kg/m^2^]	24.25 (±5.10)
WHR	0.95 (±0.10)
triceps skinfold thickness [mm]	17.50 (±7.00)
muscle mass [cm^2^]	5.41 (±2.27)
fat [%]	25.05 (±9.65)
IL-6 [pg/mL]	2.45 (±1.75)
albumin [mg/dL]	38.00 (±3.50)
prealbumin [mg/dL]	30.10 (±8.95)

BMI: body mass index; WHR: waist-to-hip ratio; SD: standard deviation; IL-6: interleukin-6.

**Table 3:** Five-year survival estimates according to different parameters (*n* = 53).

Variable	N	Level	5-Year Survival	95% C.I.	*p* Value
age (years)	16	(29, 55)	68.80	(49.40, 95.70)	0.005
	16	(55, 65)	68.70	(49.40, 95.70)	
	18	(65, 100)	44.40	(26.50, 74.50)	
sex	18	female	44.40	(26.50, 74.50)	0.404
	32	male	68.80	(54.40, 86.80)	
prealbumin (mg/dL)	29	(0, 30)	44.80	(29.90, 67.10)	0.072
	21	(30, 60)	81.00	(65.80, 99.60)	
albumin (mg/dL)	20	(0, 38)	45.00	(27.70, 73.10)	0.226
	30	(38, 50)	70.00	(55.40, 88.50)	
BMI (kg/m^2^)	23	(0, 23)	47.80	(31.20, 73.30)	0.226
	27	(23, 50)	70.40	(55.10, 89.90)	
muscle mass (cm^2^)	13	(1.88, 3.53)	30.80	(13.60, 69.50)	0.055
	14	(3.53, 5.34)	64.30	(43.50, 95.00)	
	10	(5.34, 6.83)	70.00	(46.70, 100.00)	
	13	(6.83, 13.5)	76.90	(57.10, 100.00)	
WHR	11	below limit	54.50	(31.80, 93.60)	0.226
	39	above limit	61.50	(48.00, 78.90)	
weight (kg)	13	(39.5, 61.5)	53.80	(32.60, 89.10)	0.415
	12	(61.5, 70)	41.70	(21.30, 81.40)	
	13	(70, 76.1)	61.50	(40.00, 94.60)	
	12	(76.1, 135)	83.30	(64.70, 100.00)	
fat (%)	12	(22.9, 32)	75.00	(54.10, 100.00)	0.473
	13	(8.1, 17.2)	46.20	(25.70, 83.00)	
	12	(17.2, 22.9)	66.70	(44.70, 99.50)	
	13	(32, 50.5)	53.80	(32.60, 89.10)	
IL-6 (pg/mL)	20	(0, 1.65)	60.00	(42.00, 85.80)	0.030
	7	(1.65, 1.94)	57.10	(30.10, 100.00)	
	8	(1.94, 2.52)	62.50	(36.50, 100.00)	
	11	(2.52, 8.6)	63.60	(40.70, 99.50)	

BMI: body mass index; CI: confidence interval; IL-6: interleukin-6; WHR: waist-to-hip ratio.

**Table 4:** Results of Cox’s proportional hazards analysis of survival predictors.

Variable	Adjusted HR (95% CI)	*p* Value
age [years] ref. = (29, 55)		
(55, 65)	1.28 (0.58, 2.79)	0.541
(65, 100)	5.43 (2.1, 14.07)	<0.001
prealbumin [mg/dL] (30, 60) vs. (0, 30)	0.45 (0.24, 0.84)	0.012
fat [%] ref. = (22.9, 32)		
(8.1, 17.2)	2.07 (0.81, 5.32)	0.129
(17.2, 22.9)	1.57 (0.61, 4.07)	0.351
(32, 50.5)	3.65 (1.23, 10.84)	0.019

CI: confidence interval; HR: hazard ratio; ref.: reference range.

**Table 5:** Results of a crude analysis: odds ratio for survival.

Variable	OR (95%)	*p* (Wald’s Test)
age [years] ref. = (29, 55) (55, 65) (65, 100)	1.00 (0.224, 4.459) 0.36 (0.09, 1.49)	>0.999 0.159
sex [male vs. female]	2.75 (0.83, 9.07)	0.093
prealbumin [mg/dL] (30, 60) vs. (0, 30)	5.23 (1.41, 19.43)	0.013
albumin [g/dL] (3.8, 5) vs. (0, 3.8)	2.85 (0.88, 9.26)	0.081
BMI [kg/m^2^ ] (23, 50) vs. (0, 23)	2.59 (0.81, 8.29)	0.109
IL-6 [pg/mL] ref. = (0,1.65) (1.65, 1.94) (1.94, 2.52) (2.52, 8.6)	0.89 (0.16, 5.08) 1.11 (0.21, 6.01) 1.17 (0.26, 5.33)	0.895 0.903 0.842
muscle mass [cm^2^] ref. = (1.88, 3.53) (3.53, 5.34) (5.34, 6.83) (6.83, 13.5)	4.05 (0.81, 20.2) 5.25 (0.87, 31.55) 7.50 (1.31, 43.03)	0.088 0.070 0.024
fat [%] ref. = (22.9, 9.32) (8.1, 17.2) (17.2, 22.9) (32, 50.5)	0.29 (0.05, 1.57) 0.67 (0.11, 3.93) 0.39 (0.07, 2.13)	0.149 0.654 0.277

BMI: body mass index; CI: confidence interval; OR: odds ratio; ref.: reference range.

## Data Availability

The data presented in this study are available upon request from the corresponding author. The data are not publicly available as they are still being used for analysis and manuscript writing.
